# The International Classification of Functioning as an explanatory model of health after distal radius fracture: A cohort study

**DOI:** 10.1186/1477-7525-3-73

**Published:** 2005-11-16

**Authors:** Jocelyn E Harris, Joy C MacDermid, James Roth

**Affiliations:** 1 School of Rehabilitation Sciences, University of British Columbia, T325-2211 Wesbrook Mall, Vancouver, British Columbia, V6T 2B5, Canada; 2 Rehabilitation Research Lab, GF Strong Rehab Centre, 4255 Laurel Street, Vancouver, British Columbia, V5Z 2G9, Canada; 3 School of Rehabilitation Sciences, McMaster University, Institute of Applied Health Science, 1400 Main Street West, 4^th ^Floor, Hamilton, Ontario, L8S 1C7, Canada; 4 Hand and Upper Limb Centre, St. Joseph's Health Centre, PO Box 5777, London, Ontario, N6A 4L6, Canada

## Abstract

**Background:**

Distal radius fractures are common injuries that have an increasing impact on health across the lifespan. The purpose of this study was to identify health impacts in body structure/function, activity, and participation at baseline and follow-up, to determine whether they support the ICF model of health.

**Methods:**

This is a prospective cohort study of 790 individuals who were assessed at 1 week, 3 months, and 1 year post injury. The Patient Rated Wrist Evaluation (PRWE), The Wrist Outcome Measure (WOM), and the Medical Outcome Survey Short-Form (SF-36) were used to measure impairment, activity, participation, and health. Multiple regression was used to develop explanatory models of health outcome.

**Results:**

Regression analysis showed that the PRWE explained between 13% (one week) and 33% (three months) of the SF-36 Physical Component Summary Scores with pain, activities and participation subscales showing dominant effects at different stages of recovery. PRWE scores were less related to Mental Component Summary Scores, 10% (three months) and 8% (one year). Wrist impairment scores were less powerful predictors of health status than the PRWE.

**Conclusion:**

The ICF is an informative model for examining distal radius fracture. Difficulty in the domains of activity and participation were able to explain a significant portion of physical health. Post-fracture rehabilitation and outcome assessments should extend beyond physical impairment to insure comprehensive treatment to individuals with distal radius fracture.

## Background

In 1980 the WHO [1] published a framework for classifying the consequences of disease. This classification system included the domains of impairment, disability, and handicap where a linear relationship was thought to exist between domains. This framework emphasized the multifaceted nature of health and led to changes in the measurement of health outcomes, specifically, the evaluation of disability, and handicap [2]. With increased application of the model it became apparent that the relationship between the domains was not linear and other relevant contributions to health (e.g., environmental, socio-demographic, and psychological has been ignored).

The WHO updated the framework to reflect emerging understanding of health. In 2001 the International Classification of Functioning, Disability, and Health (ICF) was published [3,4]. It has three main domains, Body Structure/Function, Activity, and Participation, that can be used to classify the impact of health. In this framework the domains interact with each other (not necessarily in a linear manner) and are influenced by both environmental and personal factors [3]. Problem areas within the domains are called impairment, activity limitation, and participation restriction. These terms decrease the negative connotations associated with earlier terminology, i.e., disability and handicap [1]. Recently, studies have linked outcome measures to the ICF domains to better reflect all aspects of health, body function, activity, and participation in musculoskeletal conditions [5-13]. With the emergence of this broader model of health, clinical research has started to focus on how ICF might explain health outcomes across a spectrum of health conditions [5-12].

Distal radius fractures are the most common fracture [14]. A 17% increase in incidence rate has been noted over the past few decades [15]. In the United Kingdom 71, 000 persons will sustain a distal radius fracture each year with an incidence rate of 36.8/10,000 for women and 8.9/10,000 for men [16]. Though distal radius fractures are found throughout the life span, women demonstrate an increase in incidence rate from age 50–70 (while men do not) which has been attributed to decreased bone mineral density [16-18].

Usually the majority of recovery from a distal radius fracture occurs within six months post fracture [19,20]. Until recently, descriptions of the clinical outcomes of distal radius fracture have focused on impairment, e.g., radiographic findings, range of motion, and strength. What are missing from these studies are outcome measures that evaluate an individual's ability to perform day-to-day tasks and engage in meaningful activities and roles. Recently studies have included broader outcome measures that reflect performance in self-care, household, work, recreational, and social activities. These studies show that despite the fact that the majority of individuals receive rehabilitation services, residual difficulty in work, sport, and leisure activities are reported [13,19,20].

Studies addressing quality of life in individuals following distal radius fracture are few. Two cross-sectional studies examined the relationship between radiographic findings and the Medical Outcomes Survey Short Form SF-36 [21] and SF-12 [22] (in long-term follow-up). Both studies found that radiographic findings did not correlate with either the SF-36 or SF-12, and that patients' post-rehabilitation scores were similar to those of the general population. However, in the study by Fernandez and colleagues [21], men between the ages of 35–44 (physical component score only) demonstrated a significant difference in SF-36 scores from their age-matched general population norm. It was suggested that this group represents a segment of the population that has greater functional demand from both work and family life and thus the health impact of mild residual physical impairment was greater.

In longitudinal studies that evaluated recovery from a distal radius fracture it is clear that health is affected in the early post-fracture period and that there is substantial recovery. MacDermid and colleagues [20] reported SF-36 scores that improved from the early post-fracture evaluation to a one-year evaluation for the Physical Component Summary Score (PCSS) (from 37–48) but found that the Mental Health Summary Component Score (MCSS) remained within normal range throughout recovery (from 51–53).

One study evaluated the adjustment to distal radius fracture over a three month time frame [23]. This study used scales that measure physical, emotional, social role function, and meaning of injury, i.e., the SF-36, the Enforced Social Dependency Scale, and the Meaning of Illness scale. Findings suggested that as time from fracture increases, scores in physical, emotional, and social role function reflect adjustment to injury [23]. The authors suggested that during the early stages of recovery significant issues in roles, physical function, and adjustment to injury are evident and should not be neglected during rehabilitation. Overall studies suggest that the impact of distal radius fracture on physical and/or mental health abates by three months post injury and occurs to a greater extent within the physical health domain as compared to mental health.

Although previous work has suggested that distal radius fracture has an impact on overall health, these studies have not focused on the extent to which health effects fit the ICH health model. An understanding of how the model applies to this common injury would assist those involved in planning or providing health services to clients with these injuries. The purposes of this study were 1) to determine whether the ICF framework serves as an explanatory model for distal radius fracture and 2) to determine the impact of impairment, activity limitation, and participation restriction on physical and mental health after distal radius fracture.

## Methods

This study used a prospective cohort design. Patients with distal radius fracture attending the Hand and Upper Limb Centre for primary care were identified by clinic lists and attending physicians. All identified patients were enrolled in the outcome evaluation process, unless they were unable to participate because of incompetence. Patients who failed or were unable to comply with their scheduled appointments were contacted by phone to determine whether they could reschedule their appointments. The university ethics review board approved the use of this clinical outcomes database for this study.

**Figure 1:**
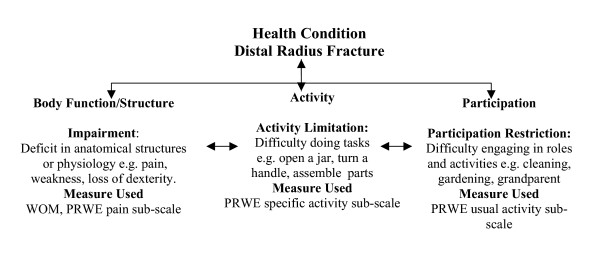
**International Classification of Functioning (ICF) model applied to distal radius fracture. **(Adapted from the World Health Organization, International Classification of Functioning, Disability, and Health training materials, Geneva, 2002). .

Patients completed standardized testing at one week, and at three and twelve months post fracture. Demographic data was collected at the initial one-week post injury visit. The ICF was used as a conceptual model to frame the health outcome of distal radius fracture. We have outlined the model, adapted from the WHO, in Figure 1.

### Outcome measures

All patients completed the Patient Rated Wrist Evaluation (PRWE) [24-26], and the SF-36 [27-29] at all 3 time points and the Wrist Outcome Measure (WOM) [30] at 3 and 12 months. A research assistant verbally administered the questionnaires (PRWE and SF-36) to patients who were unable to read or write. When patients were unable to understand English sufficiently to answer, the questionnaires were translated with the assistance of a bilingual family member or friend. All questionnaires were administered and scored according to the author's instructions. An independent research assistant administered the Wrist Outcome Measure.

The Wrist Outcome Measure is a composite impairment scale with components that reflect range of motion (ROM), grip strength, and dexterity [30]. Range of motion measures were measured on the N-K computerized hand evaluation system. A total score out of 30, scored by extent of attainment of normative values was given. Six wrist motions (extension/flexion [31], radial/ulnar deviation, pronation/supination [32]) and a gross finger flexion measure was summated. Grip strength was performed using the NK Digit-grip device. The standard protocol recommended by the American Society of Hand Therapists was followed [33]. High reliability has been demonstrated for this protocol and test instrument [34]. A grip strength (score out of 40) was determined as a ratio of the uninjured hand with the injured hand and adjusting for dominance. Dexterity was measured using the checkers subtest of the Jebson's Hand Function Test (score/15) [35]. A total Wrist Outcome Measure score out of 85 was devised from these scales. Further background and discussion on the development of an impairment rating score can be found elsewhere [30].

Patient Rated Wrist Evaluation is a 15-item questionnaire that equally rates wrist-related pain and disability in functional activities (see BMC reference for complete form) [19,24,25,36]. Scoring is done on an eleven-point scale (0–10) with zero being no issues or pain and 10 being unable to do or severe pain. There are five questions that require the individual to rate their pain doing activities such as at rest, repeated motion, and lifting. Functional items are divided into two categories, specific and usual activities. There are six specific tasks such as turning a doorknob, cutting meat, fastening a button, and four usual activity categories, self-care, work, household duties, and recreation. The PRWE can be divided into three sub-scales, pain, specific activities, and usual activities. The total of the combined scales is 100 (50 from pain, 60 from specific, and 40 from usual). The psychometric properties of this scale are excellent [19,24,25,36] and the patterns of recovery following a fracture have been described using this scale [19,20].

The SF-36 is a widely used health outcome measure. It is comprised of eight scales and two summary scores [27-29,37]. There is a large database of normative data available through the Medical Outcomes Trust. The scale has eight sub-scales that portray various domains of health: physical function, physical role, bodily pain, vitality, general health, emotional role, mental health, and social function. These sub-scales are scored out of a maximum score of 100 (higher is better). The physical and mental health component summary scores represent the two main dimensions of health. These scores are calculated in a three-step process which involves weighting, transforming and aggregating the subscale scores to compute summary scores scaled to a US population which represent these two distinct domains of health (US population mean = 50). While the ICF model portrays health as a single concept with multiple, interacting contributors, the SF-36 separates physical and mental health. Since we expected largely a physical effect of wrist fracture and because the SF-36 has been shown to be preferable to other general health measures for musculoskeletal disorders [38,39] we choose it to represent health status. While lesser effects were expected in mental health we decided to include both the Mental and Physical Component Summary Scores as outcomes to determine the relative effects on both domains of health providing a more complete picture of overall health.

### Data analysis

Descriptive statistics were calculated for the dependent variables (SF-36 physical and mental health component summary scores) and independent variables (WOM total score, PRWE sub-scales pain, specific activities, and usual activities). All data was inspected for assumption violation by using histograms, box, and scatter-plots. Missing values were replaced using linear extrapolation. Missing values accounted for less than 5% of data points.

Univariate analysis was completed to determine the relationship between variables of interest and outcome variables. Pearson's Correlation Coefficient was used to determine the relationship between SF-36 physical and mental health summary scores and the PRWE pain, specific, and usual activity scales, and the WOM total score. Correlation was determined at time one (one week post injury, time two (three months post injury), and time three (twelve months post injury).

Multivariate analysis was used to determine the explanatory model for distal radius fracture health outcome at time one (one week post), two (three months post), and three (twelve months post). Multiple regression equations were calculated using the SF-36 physical and mental health summary scores as the dependent variables, and patient characteristics, PRWE sub-scales and the WOM as the independent variables as described in Figure 1. The WOM was measured at three and twelve months but not at one week. It was felt that the variables of sex and age are known to be related to health, so we controlled for age and sex by blocked entry of these variables and then continued with stepwise entry of the independent variables. Six stepwise regression models were built. Data was inspected for assumption violation by examining box and scatter-plots of residuals against explanatory variables from each model. Influential data points were examined using Cook's distance. The F to enter was 0.05 and the F to remove was 0.10. Statistical significance was set at 0.05 for all outcomes. All statistics was performed using SPSS 13.

## Results

### Sample characteristics

There was a total of 790 persons, mean age of 51.4 (SD = 17.6, age range 18–91) in this study. The majority of the people in the study were female (68%). Descriptive characteristics of the sample can be found in Table 1. Summary scores for the outcome measures at each time frame can be found in Table 2. The mean score of the outcome measures improve at each follow up time period. At one week post injury scores demonstrate moderate to severe activity limitation and participation restriction and the one-year measures demonstrate little activity limitation or participation restriction. The PRWE specific activity sub-scale showed the most change over time, from 51.3 (severe limitation) to 6.3 (minimal limitation).

**Table 1:** Sample characteristics

Variable	Description
Sex	Male = 251
	Female = 539
Dominant Hand	Right = 90%
	Left = 10%
Wrist Injured	Right = 45%
	Left = 49%
Mechanism of Fracture	Fall on ice = 18%
	Other fall = 66%
	Other = 10%
Energy of Fracture*	Low = 69%
	Medium = 19%
	High = 6%
Highest Level of Education	Finished high school = 26%
	Finished college = 18%
	Finished university = 8%
	Finished graduate school = 4%
Occupation at Injury†	Retired = 27%
	Service = 13%
	Professional = 12%
Occupational Demand§/P >	Low = 57%
	Moderate = 24%
	High = 19%
Had Physiotherapy	83%

* Low = fall from a standing position, Medium = fall from a height, High = trauma

† Top three occupations

§Self-report of how much they used their hand at work; Low = low force, low repetition, Moderate = frequent repetition, intermittent force, High = high force, constant repetition

**Table 2:** Descriptive statistics for outcome measures at one week, three months, and one year post injury.

Variable	**Time 1 Mean (SD)**	**Time 2 Mean (SD)**	**Time 3 Mean (SD)**
Wrist Outcome Measure (/85; 85 = best)	N/A	59.6(8.9)	73.9(7.4)
PRWE pain scale (/50; 50 = worst)	30.2(11.6)	17.0(10.4)	8.1(9.5)
PRWE specific scale (/60; 60 = worst)	51.3(14.1)	19.4(15.0)	6.3(10.3)
PRWE usual scale (/40; 40 = worst)	26.3(11.9)	11.4(12.5)	5.6(12.6)
SF-36 physical health (US norm 50)	37.2(8.7)	43.7(8.9)	49.0(8.7)
SF-36 mental health (US norm 50)	49.8(11.2)	51.5(9.8)	54.8(7.5)

### Univariate analysis

Results from the univariate analysis can found in Table 3. All PRWE sub-scales were correlated with SF-36 physical health at one week, three, and twelve months post injury. At one week the sub-scale of usual activity demonstrated the highest correlation (r = -0.31, p = 0.01), at three and twelve months it was specific activity (r = -0.53, p = 0.01, r = -0.52, p = 0.01). Only usual activity was correlated with mental health (r = 0.09, p = 0.05) at one-week post injury. However, at three months all PRWE sub-scales were significantly correlated to physical health and at twelve months all independent variables (PRWE and WOM) were significantly correlated.

**Table 3:** Correlation results between outcome measures at one week, three months, and one year post injury.

**Variable**	**PRWE pain**	**PRWE specific**	**PRWE usual**	**SF-36 physical health**	**SF-36 mental health**
Wrist Outcome Measure					
Time 2	-0.27**	-0.35**	-0.20**	0.21**	0.07
Time 3	-0.43**	-0.46**	-0.44**	0.32**	0.14**
PRWE pain					
Time 1		0.46**	0.44**	-0.27**	0.05
Time 2		0.75**	0.53**	-0.50**	-0.27**
Time 3		0.79**	0.34**	-0.50**	-0.34**
PRWE specific					
Time 1			0.48**	-0.29**	0.01
Time 2			0.57**	-0.42**	-0.23**
Time 3			0.34**	-0.52**	-0.30**
PRWE usual					
Time 1				-0.31**	0.09*
Time 2				-0.42**	-0.26**
Time 3				-0.16**	-0.10**
SF-36 physical health					
Time 1					-0.002
Time 2					0.10**
Time 3					0.13**

* p = 0.05, **p = 0.01

† Outcome variables are correlated with each variable at their respective time periods, i.e., time 1 with time 1, time 2 with time 2, etc.

### Multivariate analysis

All regression results can be found in Tables 4, 5, and 6. The result from the forward stepwise regression model for SF-36 physical health at one-week post injury yielded a weakly predictive model where all PRWE sub-scales were retained with an R^2 ^= 0.13, p < 0.0001 for the full model (Table 4). Usual activity was most predictive and accounted for R^2 ^= 0.10 of the model. For SF-36 mental health, minimal effects were observed with only PRWE usual activity retained within the model R^2 ^= 0.01, p = 0.04.

**Table 4:** Multiple regression results for SF-36 Physical Health one week post injury

**Variable**	**R^2^**	**Standardized Beta**	**P value**
**SF-36 Physical Health Summary Scale, Total Model R^2 ^= 0.13, p < 0.0001**			
PRWE usual*	0.10	-0.19	0.0001
PRWE specific	0.12	-0.15	0.0001
PRWE pain	0.13	-0.12	0.003

* Independent variable scores are from one-week post injury

**Table 5:** Multiple regression results for SF-36 Physical and Mental Health three months post injury

**Variable**	**R^2^**	**Standardized Beta**	**P value**
SF-36 Physical Health Summary Scale, Total Model R^2 ^= 0.33, p < 0.0001*			
PRWE specific†	0.28	-0.27	0.0001
PRWE pain	0.31	-0.23	0.0001
PRWE usual	0.33	-0.15	0.0001
**SF-36 Mental Health Summary Scale, Total Model R^2 ^= 0.10, p < 0.0001§**			

PRWE pain	0.08	-0.18	0.0001
PRWE usual	0.10	-0.17	0.0001

* Excluded variable Wrist Outcome Measure

† Independent variable scores are from three months post injury

§Excluded variables Wrist Outcome Measure, PRWE specific

**Table 6:** Multiple regression results for SF-36 Physical and Mental Health one year post injury

**Variable**	**R^2^**	**Standardized Beta**	**P value**
SF-36 Physical Health Summary Scale, Total Model R^2 ^= 0.28, p < 0.0001*			
PRWE specific†	0.25	-0.29	0.0001
PRWE usual	0.28	0.28	0.0001

* Excluded variables PRWE pain and Wrist Outcome Measure

† Independent variable score are from one-year post injury

The regression results for three-month post injury models yielded greater prediction form included variables (Table 5). All PRWE sub-scales were retained in the SF-36 physical health model with a total R^2 ^= 0.33, p < 0.0001. PRWE specific activity was most predictive and accounted for R^2 ^= 0.28 of the total model. Again at the three-month model, SF-36 mental health was less explained by wrist scores, but the independent variables PRWE pain and usual activity were retained, R^2 ^= 0.10, p < 0.0001. PRWE pain accounted for the major effect, R^2 ^= 0.08 of the total model.

The regression results for one-year post injury can be found in Table 6. At one year post injury the regression model for SF-36 physical health showed that PRWE specific and usual activity accounted for a total R^2 ^= 0.28, p < 0.0001, with specific activity accounting for the majority of the model, R^2 ^= 0.25. The regression model for SF-36 mental health one year post injury showed that only PRWE pain was retained and accounted for a total R^2 ^= 0.08, p < 0.001.

## Discussion

This study determined that the ICF framework is supported when evaluating the impact of distal radius fracture on health because impairment, activity limitations and participation restrictions, individually and in combinations, were related to self-reported physical health status as measured on the SF-36. Statistically significant models for SF-36 physical and mental health were found. The models for SF-36 physical health were strong with the PRWE accounting for 13% (one week), 33% (three months), and 28% (one year) of the variance. Models for SF-36 mental health demonstrated weak relationships with the PRWE accounting for 1% (one week), 10% (three months), and 8% (one year) of the variance. This study confirms previous work that a distal radius fracture mainly affects the physical domains of health, although it does suggest that pain levels and mental health are also related.

The ICF framework is advantageous as the inclusion of aspects relating to the injury, the individual and the environment provide a broader view of how health interventions might be undertaken. In the past the focus of outcome for distal radius fracture has been impairment based (e.g., radiographic data, strength, and range of motion). However, studies have shown that impairment is not necessarily the best method to measure outcome as it does not always reflect activity and or participation restrictions [13,22,40]. We measured health by self-report allowing us to capture early and late health effects. The PRWE sub-scales were able to explain a significant portion of the SF-36 physical health score at all time periods. When examining the areas measured by the PRWE sub-scales this indicates that problems in areas such as pain, dexterity, lifting, work, household duties, recreation, and self-care after fracture do contribute to overall physical health. However, there were differences in the magnitude of the models and the most prevalent sub-scale between the time periods suggesting that impairment, activity, and participation have different health impacts at different time points in recovery from distal radius fracture.

The regression equations at one-week explained relatively little of the health impact of the fracture. This may have been because PRWE scores are consistently very poor at this point across all patients [19], and variations in health status were not well reflected at this point. For example, many patients would be immobilized providing a common restriction on specific activities like lifting or getting up from a chair. The PRWE usual activity focused on ability to do usual activities and was significant, although accounting for only 10% of the variance of the physical health model (R^2 ^= 0.13) at one-week post injury. This sub-scale evaluates perform their usual self-care, work, household duties, and recreational activities (i.e., participation). This suggests that there is more variability in ability to participate in usual activity, than is observed on the specific activities subscale and these variations impacted on health status. This is in agreement with qualitative studies where clients emphasized the impact of distal radius fracture on work and household activities [41,42]. It is worth noting that issues with pain and inability to do specific activities were not unimportant at this time as they were rated as being at high levels of limitation. Given that participation in usual activity was related to early health status it might be worthwhile to provide some focus on methods to adapt to limitations in the early stage of fracture treatment. Individuals might benefit from educational materials that outline expected activity limitations and possible adaptations to maximize their ability to perform common tasks of daily life.

SF-36 physical health scores at three months and one-year post injury were explained to a greater extent by variations in the PRWE. The PRWE sub-scale of specific activity explained between 25% (model R^2 ^= 0.28) and 28% (model R^2 ^= 0.33) of the variance, replacing usual activity as the prominent variable. At three months post injury the cast has been removed and rehabilitation is underway and patients may be variable in their inability to do specific tasks that require pain-free wrist motion or strength. Self-reported wrist/hand activity limitations and participation restrictions explained a significant portion of overall physical health scores indicating that the wrist injury has a substantial impact on overall health in a manner that is consistent with the view of health portrayed in ICF. Composite wrist impairment rating had minimal additional influence on the models accounting for between 1% and 3% additional variance in health. This is in agreement with the studies that found that impairment was not a good indicator of function [13,21,22,30,40]. Physical impairment contributes to activity limitation, however, there is not a direct relationship.

Though the PRWE sub-scales accounted for a significant portion of the SF-36 physical health score (13%-33%), there are obviously other influencing factors that remain unexplained. We were unable to address a broad spectrum of potentially useful variables given database limitations. More complex models are needed to explore the remainder of the variance and additional concepts such as workplace environment; rehabilitation, surgery, socio-economic status, etc., should be included.

Physical and mental health domains have been seen as distinct domains of health according to SF-36 development and validation. This was also true in this study where the correlation between the physical and mental component summary scores was very low (r<0.10). Neither physical impairments, activities limitations nor participation restrictions were strongly predictive of SF-36 mental health scores. This is consistent with the view that distal radius fracture primarily affects physical health. The PRWE pain sub-scale accounted for slight variation in SF-36 mental health scores. Pain however, has not been shown to significantly impact recovery of distal radius fracture [43,44]. In fact in a study by Bialocerkowski [42], clients with wrist disorders were asked to explore difficulties post injury and pain was not mentioned. Instead issues with household duties, work demands, recreation, and fine motor skills were identified. Because regression reveals associations, it is not clear whether higher pain lowers mental health, or if those with poorer mental health experience more pain.

In describing rehabilitation following distal radius fracture, the importance of a staged approach has been suggested [45-47]. This study would support a staged approach as the mediators of physical health vary over time. In the early phases where immobilization and fracture healing limit motion and activity, the ability to perform usual activities is important. In addition to the customary attention to pain management, rehabilitation should also include compensatory strategies or aids as required to assist individuals complete their usual activities. In the rehabilitative phase where ability to perform specific tasks plays a larger role, remediation of impairment, incorporation of client driven goals and activity-based rehabilitation [48] are needed. Finally, the importance of participation is highlighted in these models and suggests that participation in usual self-care, household, work, and recreation, must be maximized to restore physical health. It has been demonstrated that even after adjusting for age and comorbidity patients more than 65 years of age who sustain a wrist fracture have a 57% 7-year survival rate, as compared to 71% for the comparative US population [49]. One possible contributor to this problem can be reduced participation in an active lifestyle that increases risk for additional health problems. Addressing participation during fracture rehabilitation may have short-term and longer-term health benefits.

## Conclusion

The ICF model is useful in framing the health effects of a distal radius fracture that has implications for optimal management of distal radius fractures. Self-reported health measures should provide insight into the impairment, activity limitations and participation restrictions that result from a distal radius fracture. These aspects of health can be addressed at different phases of fracture management and rehabilitation to provide optimal physical health recovery.

## Authors' contributions

JM formed the original study design and clinical database, obtained the ethics approval and obtained database funding. JH and JM developed the research question and planned statistical analyses. JH conducted statistical analyses and drafted the manuscript. JR enrolled, treated and evaluated patients in this study and contributed to the design of evaluation procedures, study equipment and personnel funding. All authors approved the final study protocol, contributed to interpretation of the study results and participated in revisions of the manuscript. All authors read and approved the final manuscript.
